# Feasibility of T2 Mapping and Magnetic Transfer Ratio for Diagnosis of Intervertebral Disc Degeneration at the Cervicothoracic Junction: A Pilot Study

**DOI:** 10.1155/2019/6396073

**Published:** 2019-05-02

**Authors:** Chao Zhang, Yuan Lin, Zhihua Han, Liang Gao, Ruipeng Guo, Qinglei Shi, Lei Chen, Chun Chen

**Affiliations:** ^1^Department of Orthopaedic Surgery, The First Affiliated Hospital of Wenzhou Medical University, Wenzhou 325000, China; ^2^Department of Sports Medicine and Arthroscopic Surgery, The First Affiliated Hospital of Anhui Medical University, Hefei 230000, China; ^3^Experimental Trauma & Orthopedic Surgery, Frankfurt Initiative for Regenerative Medicine, JW Goethe-University, Frankfurt am Main 60528, Germany; ^4^Center of Experimental Orthopaedics, Saarland University, Homburg/Saar 66421, Germany; ^5^Siemens Ltd., China Healthcare Sector MR Business Group, 100102, Beijing, China; ^6^Engineering Research Center of Clinical Functional Materials and Diagnosis & Treatment Devices of Zhejiang Province, Wenzhou Institute of Biomaterials and Engineering, 325001, Wenzhou, China

## Abstract

**Background:**

Intervertebral disc degeneration (IDD) at the cervicothoracic junction of spine is clinically relevant, however, little attention had been paid. T2 mapping and magnetic transfer ratio (MTR) are useful magnetic resonance imaging (MRI) techniques to quantitatively evaluate IDD, revealing the biochemical changes within the intervertebral disc. To compare T2 mapping with MTR imaging regarding their accuracy to quantitatively diagnose intervertebral disc degeneration at the cervicothoracic junction, influences of anatomical level, gender, age, and Pfirrmann grade of T2 relaxation time values and MTR values were evaluated.

**Methods:**

Sixty-seven patients with neck and upper back pain were included and examined with both T2 mapping and MTR imaging. The Pfirrmann grade, T2 relaxation time values, and MTR value of each disc between C7 and T3 were measured. Differences were investigated among different segmental levels, genders, age ranges, and Pfirrmann grades. The diagnostic accuracy of both MRI techniques was compared using the receiver operating characteristic (ROC) curves.

**Results:**

No significant difference was detected comparing T2 relaxation time values or MTR values among different anatomical levels, genders, and segmental levels. And we generally found that T2 relaxation time values decreased, while MTR value increased with increasing age. Importantly, we demonstrated the significant correlation between either T2 relaxation time values or MTR value and Pfirrmann grade.

**Conclusion:**

We proved the better accuracy of T2 mapping over MTR imaging to quantitatively evaluate the intervertebral disc degeneration of the cervicothoracic junction.

## 1. Introduction

The neck pain and upper back pain, attributed to intervertebral disc degeneration (IDD) of the cervical spine, are common complaints in outpatient department. As recently reported, disc degeneration at cervicothoracic junction, 7th cervical (C7) to the 3rd thoracic (T3) vertebrae, could also bring as similar symptoms as cervical disc degeneration [1]. In particular, cervical disc herniation owns approximately 4% to 8% to disc herniation at C7-T1 [2, 3], while disc herniation at T1-T2 [4–6] and T2-T3 [7–9] is also noted. However, little attention has been paid to the radiographic diagnosis of the disc degeneration at this clinical relevant region.

Quantitative magnetic resonance imaging (MRI) techniques have been applied to detect intervertebral disc degeneration, especially at its moderate stage by associating MRI signal with biochemical components of the nucleus pulposus (NP) and annulus fibrosus (AF) [10–13]. Better than semiquantitative morphological MRI techniques, such as traditional T2-weighted imaging, quantitative MRI techniques showed higher accuracy [14–16].

Among those newly emerging quantitative MRI techniques, T2 mapping and MTR (magnetization transfer ratio) imaging have recently received considerable clinical attention. Specifically, T2 mapping measures T2 relaxation time values, which reflects the content of water and proteoglycans (PG); higher disc water or PG content results in increased T2 relaxation time values, whereas more collagen content contributes to shorter T2 relaxation time values [12]. Our previous study also demonstrated that T2 values of the NP associated with Pfirrmann grades [1] at C2-T1 region, exhibiting an inverse correlation between the T2 relaxation time values and the Pfirrmann grade, of which method is widely used to morphologically rate disc degeneration [17].

Additionally, cartilage has been demonstrated to exhibit significant magnetization transfer (MT) effect under particular magnetization stimulus, attributing to collagen, the predominant macromolecular component of cartilage [15]. MT effect is displayed on the basis of two different proton pools [18]: the bound proton pool that protons bound to macromolecular. These protons are not visible with normal MRI because of their restricted mobilities; the unbound (free water) pool is visible through common MRI. These two proton pools are biochemically and magnetically equivalent in many types of human tissues, like muscle and cartilage. However, after using off-resonance pulses to saturate the magnetization of bound water molecules, such equilibrium is moved to the bound proton pool and the observable magnetization will reduce, leading to a reduction of the MR signal. The resulting signal attenuation in the mobile pool gives a signal reduction by imaging the pulse sequence with MT pulse. In two independent imaging sequences with MT (Ms) and without MT (Mo), a saturation pulse, the magnetization transfer can be measured as an MT ratio (MTR) calculated voxel by voxel: MTR = (Mo−Ms)/Mo in percentage [19–21].

Furthermore, stem-cell-based therapies are emerging as promising alternatives to treat intervertebral disc diseases [22–24], which is considered to be more effective in the mildly and moderately symptomatic degenerated discs (Pfirrmann grade II-III) rather than severely degenerated ones (Pfirrmann grade IV-V) [23]. Furthermore, the outcomes of these newly emerged therapies can be monitored by investigating the biochemical changes within the treated discs, which is unable to be achieved with traditional MRI techniques. Thus, more sophisticated MRI techniques with improved accuracy are clinically necessitated to quantitatively identify the intervertebral disc degeneration and to supervise the outcomes of cell-based therapies.

The aim of this pilot study was to evaluate T2 mapping and MTR imaging for the quantitative diagnosis of the intervertebral disc degeneration at the cervicothoracic junction. Influences of specific factors (anatomical level, gender, age, and Pfirrmann grade) and the diagnostic accuracy of both techniques were investigated.

## 2. Materials and Methods

### 2.1. Subject Selection

Approval for this study was obtained from the Ethics Committee of the First Affiliated Hospital of Wenzhou Medical University, Wenzhou, China. Written and informed consent was obtained from all participants. The patients were enrolled when they met the following criteria [25]: (1) main complaint of neck and upper back pain, including neck or arm weakness, tingling, or numbness; (2) these sufferings are severe enough for the patients to seek medical remedy [1]. Also, subjects were excluded if they had (1) systematic diseases (e.g., diabetes mellitus, osteoporosis, and tumors), (2) history of spinal and back surgery, and (3) implants contraindicated by the MRI unit.

### 2.2. Magnetic Resonance Image Acquisition

All MRI examinations of the cervicothoracic junction were conducted with a 3.0 Tesla MR scanner (Magnetom Skyra, Siemens Healthcare, Erlangen, Germany) in the afternoon to minimize the diurnal variation of water content within the intervertebral discs. Five MRI sequences were applied consecutively (Table S1): T1-weighted imaging (T1-WI), T2-weighted imaging (T2-WI), no-MT and MT imaging, and T2 mapping. Specifically, the sagittal T1-WI and T2-WI fast spin echoes were used to morphologically evaluate the IVDs of interest based on Pfirrmann's method [17].

Then, the T2 relaxation time values of each disc were measured on the sagittal T2 mapping image built on the mid-sagittal section centered on the midline of the cervical spine. Such T2 mapping image was optimized when an eight-multispin echo sequence was applied. The signal intensity (SI) of the T2 maps was computed on a pixel-by-pixel basis according to the formula for each respective TE: SI = e^–TE/T2^ [20, 26].

Following MTR imaging that was performed to each subject, the MT effect was detected using a sagittal gradient echo sequence (TR/TE:107/8.0 milliseconds) with dual signal acquisition: with MT prepulse that an off-resonance pulse applied at 1100 Hz down to the free water proton resonance frequency (Ms) and without MT prepulse (Mo) [21].

### 2.3. Image Processing and Analysis

**Figure 1:**
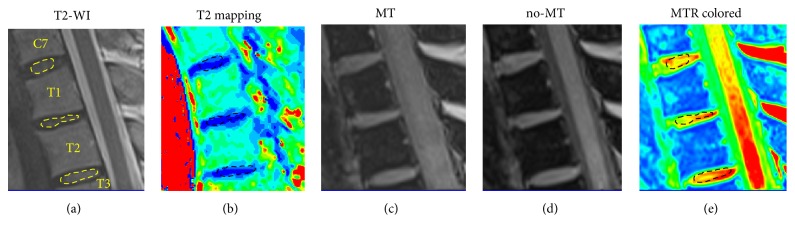
Representative images showing the series of MRI evaluations of the cervicothoracic junction and the placement of ROIs. (a) The T2-weighted MR image of the cervicothoracic junction and the ROIs were manually drawn to include the NP tissue of the discs. (b) T2 mapping image based on the T2 relaxation time value. (c) MTR image that was obtained when the magnetic pulse was applied. (d) No-MT image that was obtained without applying magnetic pulse. (e) MTR colored image according to the MTR values calculated from MT image and no-MT image.

Two radiologists, each with more than 10-year experience in the musculoskeletal radiology, independently evaluated the intervertebral disc degeneration and morphologically graded all discs in consensus according to the Pfirrmann grading system according to the height of the disc, the change of intensity of the nucleus pulposus, and the distinction between the nucleus and the annulus [17]. An orthopedic surgeon with more than 5-year experience of spine MR imaging processed and analyzed T2 mapping and MTR images on a dedicated workstation (Syngo Multimodality Workplace, Erlangen, Germany). The values of T2 relaxation time and MTR of the intervertebral discs were obtained through meticulously placing the regions of interest (ROI) on the nucleus pulposus. To maximize the accuracy of ROI placing, a free-hand tool was utilized to draw the ROIs to define the inner part of each disc [1, 27]. In particular, the ROIs were manually and carefully drawn to accord with the shape of the nucleus pulposus shown on the middle slice of all sagittal T2-WI images (Figure 1) and then copied to T2 mapping and MTR images at the same anatomic position (Figure 1).

### 2.4. Comparison of T2 Relaxation Time Values and MTR Values

Results of T2 relaxation time values and the MTR values were compared based on different anatomical level, gender, age ranges (20-29, 30-39, 40-49, 50-59, and ≥60 years), and Pfirrmann grades. Additionally, the correlations between the T2 relaxation time values and the MTR values and Pfirrmann grades were evaluated separately.

### 2.5. Inter- and Intraobserver Analysis by ROC Curves

To determine the diagnostic accuracy of intervertebral disc degeneration grading, we plotted receiver operating characteristic (ROC) curves to investigate the sensitivity and specificity of both the T2 relaxation time values and the MTR value in assessing the intervertebral disc degeneration at each Pfirrmann grade. The area under the curve (AUC) was calculated for each curve, while the cut-off values were determined by choosing the points indicating the maximum “sensitivity and specificity” values as described before [28].

### 2.6. Statistical Analysis

The results are presented as mean ± standard deviation (SD). The SPSS 24.0 (SPSS, Chicago, Illinois, USA) software was used to conduct all statistical analyses. Student's *t*-test and one-way analysis of variance (ANOVA) were used to compare the T2 relaxation time values and MTR values between different anatomic levels, genders, age ranges, and Pfirrmann grades. Moreover, the correlations of T2 relaxation time values or MTR values with either age or Pfirrmann grade were evaluated by Spearman rank correlation analysis. P < 0.05 was considered as statistically significant.

## 3. Results

### 3.1. Morphological MRI Findings

This study included 67 symptomatic subjects (35 males and 32 females; age ranged from 24 to 78). And the numbers of patients of constructive age ranges were 19 (20-29 years old), 8 (30-39 years old), 14 (40-49 years old), 12 (50-59 years old), and 14 (older than 60 years old), respectively. And 201 IVDs without susceptibility artifacts were used to measure the T2 relaxation time values and MTR values and then quantify the degeneration of the cervicothoracic junction. Particularly, as shown in Table 1, 32 discs at C7-T1 exhibited mild or moderate degeneration (grade II-III), while 18 discs presented severe degeneration. Similarly, 32 discs at T1-T2 were with grade II-III degeneration and 16 discs with severe degeneration. At the level of T2-T3, mild or moderate degeneration was shown in 40 discs, and only 6 discs showed severe degeneration.

### 3.2. T2 Relaxation Time Values and MTR Values of Different Segmental Level

**Figure 2:**
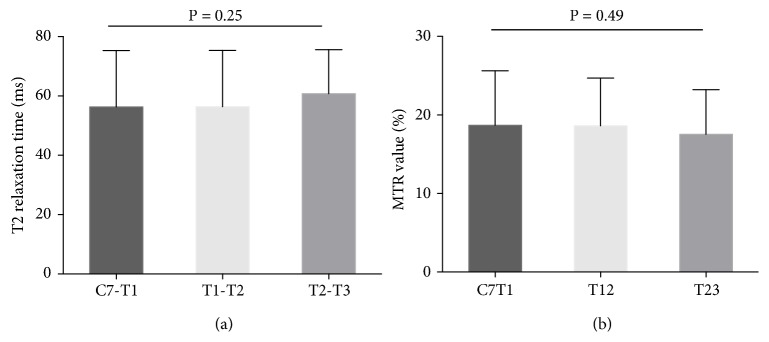
T2 relaxation time values and MTR values at different segmental levels. (a) No significant difference of the T2 relaxation time values at different levels was detected (P = 0.25, by one-way ANOVA). (b) MTR values of three anatomical levels showed no significant difference (P = 0.49, by one-way ANOVA).

As shown in Table 2, the T2 relaxation time values and MTR values were presented according to the segmental level and sex. For different levels, the T2 relaxation time values at C7-T1,   T1-T2, and T2-T3 were 56.29 ± 19.04 ms, 56.37 ± 18.97 ms, and 60.78 ± 14.83 ms, respectively. The MTR values at C7-T1, T1-T2, and T2-T3 were 18.68 ± 6.94, 18.62 ± 6.09, and 17.53 ± 5.70, respectively. We found no significant difference of either T2 relaxation time values or MTR values among different anatomical levels (P = 0.25 and P = 0.49, resp.; one-way ANOVA; Figure 2).

### 3.3. T2 Relaxation Time Values and MTR Values of Different Gender

We found no significant difference when we compared the T2 relaxation time values or the MTR values between males and females at each level (Table 2).

As to the T2 relaxation time values, the females showed similar mean values to males without statistical significance at all segmental levels (Table 2). Similarly, such trend was also exhibited regarding the MTR values; the female patients had approximate mean ADC values at all anatomical levels.

### 3.4. T2 Relaxation Time Values and MTR Values of Different Age Ranges

The T2 relaxation time values decreased as the age increased, while MTR value increased with increased age. The T2 relaxation time values declined from 75.78 ms at the age of 20-29 to 37.10 ms at the age above 60 (Table S2). Likewise, the MTR values increased from 11.37 % at the age of 20-29 to 25.98 % at the age above 60.

**Figure 3:**
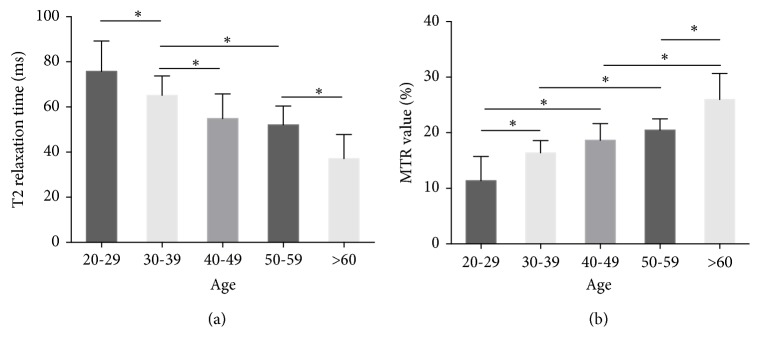
T2 relaxation time values and MTR values at different age. (a) The T2 relaxation time values decreased evidently with the increased age. (b) The MTR values generally increased with the increasing age. *∗*P < 0.05. All P values of multicomparisons were calculated by post hoc test of one-way ANOVA.

With one-way ANOVA analysis, we found that statistical significances existed in the comparison of T2 relaxation time values between each two neighbor age ranges, except between 40-49 and 50-59. In particular, the mean value of T2 relaxation time values was 54.86 ± 8.35 ms at the age of 40-49 years and slightly decreased to 52.06 ± 8.35 ms at the age of 50-59 years without statistical significance being reached (P > 0.05). Likewise, we also found that MTR values increased with increasing age and the statistical significances were detected between every two age ranges, except 30-39 versus 40-49 and 40-49 versus 50-59 (Figure 3 and Table S2). Specifically, the MTR mean value increased from 16.38 ± 2.20 (%) at age of 30-39 years to 18.65 ± 3.00 at age of 40-49 and then to 20.47 ± 2.01 (%) at age of 50-59 years with no statistically significant difference (P > 0.05).

### 3.5. T2 Relaxation Time Values and MTR Values of Different Degeneration Grade

**Figure 4:**
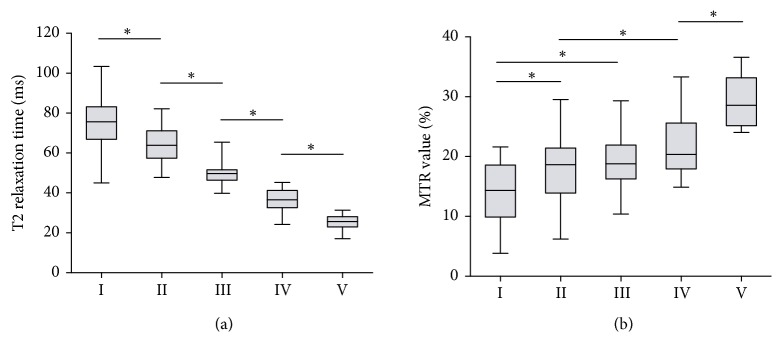
T2 relaxation time values and MTR values of different Pfirrmann grades. (a) The T2 relaxation time values declined from grade I to grade V with statistical significance existing in the comparison of each two adjacent Pfirrmann grades (all P < 0.05). (b) The MTR values increased with the advance of the Pfirrmann grade without detecting significant differences between grade II and III and between grade III and grade IV. *∗*P < 0.05. All P values of multicomparisons were calculated by post hoc test of one-way ANOVA.

T2 relaxation time values tended to decrease and the MTR value increased with the advance of the Pfirrmann grade. A significant negative correlation was observed between the T2 relaxation time values and the Pfirrmann grade, and a positive correlation was found between the MTR value and the degenerative severity. Particularly, the T2 relaxation time values decreased from 76.44 ± 12.25 ms at grade I to 25.35 ± 3.85 ms at grade V with statistically significant differences between each two adjacent grades (all P < 0.05) (Figure 4 and Table S3).

Moreover, a significant negative correlation between the T2 relaxation time values and the degeneration grade was detected (r = -0.87, P < 0.001) by Spearman rank correlation analysis. Likewise, the MTR value showed a growth from 13.93 ± 5.07 (%) at grade I to 29.00 ± 4.09 (%) at grade V. Being contrary to T2 relaxation time values, the MTR value was positively correlated to the Pfirrmann grade (r = -0.62, P < 0.001) (Figure 4 and Table S3). Interestingly, although significances existed when comparing the MTR value of either grade IV or grade V with that of grade I or grade II (all P < 0.05), multicomparison tests detected no significant difference of the MTR value between grades II and III and between grade III and grade IV (all P > 0.05).

### 3.6. Diagnostic Accuracy of T2 Mapping and MTR

**Figure 5:**
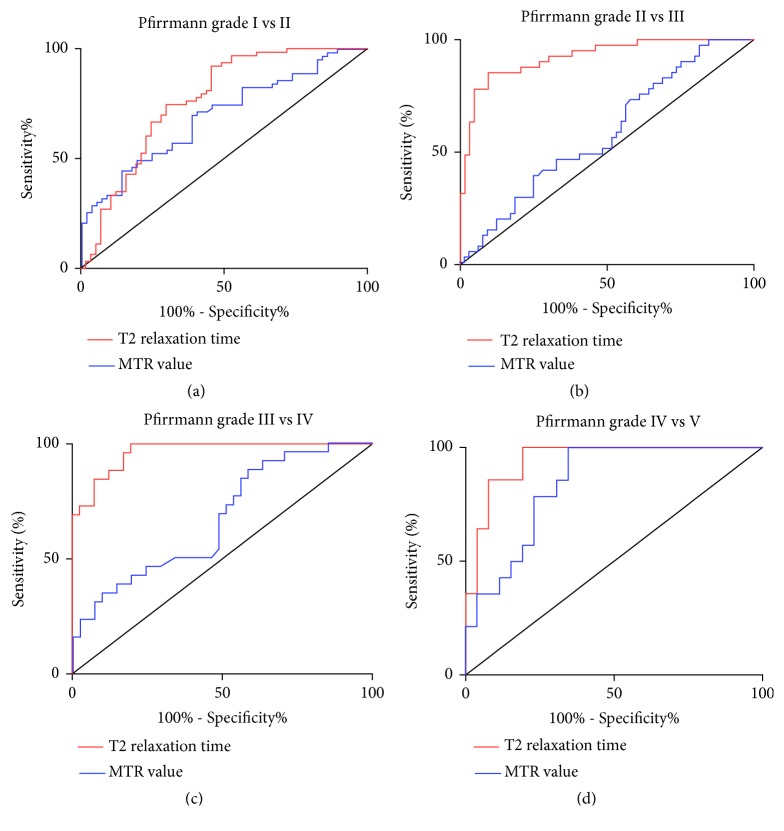
Receiver operating characteristic (ROC) curves to compare the diagnostic accuracy of T2 mapping and MT imaging. (a) The ROC curves comparing T2 relaxation time values and MTR value of grade I* versus* II showed larger area under curve (AUC) of T2 relaxation time value (0.77) than that of MTR value (0.69). (b) The ROC curves of grade II* versus* III. Similarly, the AUC of T2 relaxation time value was larger than that of MTR value (0.92 and 0.58, resp.). (c) The ROC curves of grade III* versus* IV. The AUC of the curves of T2 relaxation time value and MTR value were 0.97 and 0.67. (d) The ROC curves of grade IV* versus* grade V. The AUC of the curve of the T2 relaxation time values (0.94) was approximate to that of the curve of the MTR value (0.84).

The ROC curves analysis demonstrated that T2 relaxation time values obtained better sensitivity and specificity than MTR regarding quantitatively diagnosing the disc degeneration of the cervicothoracic junction. Mainly, the T2 relaxation time values allowed for quantitative distinguishing of disc degeneration of all Pfirrmann grades (I-V), which was unable to be achieved with the MTR values (Figure 5 and Table S4). The T2 cut-off values between each of two advancement Pfirrmann grades were selected to be 77.12, 56.40, 45.45, and 31.70 ms, respectively (Table S4). The AUC values of all ROC curves of T2 mapping ranged from 0.77 to 0.97 (Figure 5 and Table S4), and all T2 relaxation time values and cut-off values were yielded with their sensitivities from 85.37 % to 100% and specificities from 47.37% to 90.48%.

Regarding the MTR values, the cut-off values between each of two consecutive Pfirrmann grades were chosen to be 15.96%, 12.17%, 17.24%, and 23.78%, respectively (Table S4). The AUC values of all ROC curves of the MTR values ranged from 0.58 to 0.84 (Figure 5 and Table S4), and all MTR cut-off values were yielded with their sensitivities from 69.84% to 100% and specificities from 17.46% to 65.38%. Generally, the T2 relaxation time values obtained higher AUC values of the ROC curves than the MTR value, especially for differentiating grades II and III (0.92 versus 0.58) and grades III and IV (0.97 versus 0.67) (Table S4). Moreover, the sensitivity and specificity of the T2 relaxation time values were also higher than those of the MTR value at distinguishing each two neighbor Pfirrmann grades.

## 4. Discussion

The most significant finding of the present study was that T2 mapping possessed a better accuracy than MTR imaging for quantitatively diagnosing the IDD at the cervicothoracic junction. Furthermore, we demonstrated that the T2 relaxation time values negatively correlate to the disc degeneration, while MTR values positively correlate to the Pfirrmann grades at this region.

In this study, the cervicothoracic junction was defined to be C7-T1, T1-T2, and T2-T3 and their attachments of the paraspinal soft tissue. The concept of the cervicothoracic junction is still debatable, and few studies had been conducted to understand the disc degeneration of this region. It was defined by Miscusi as the region from the C7 to T4 and their attachments [29], yet others considered the cervicothoracic junction as the C7 and T1 vertebrae, the disc between C7-T1, and the attachments of the paraspinal soft tissue [2, 30].

In spite of such disagreement, the disc degeneration at this critical area is as inevitable as other parts of the spine and an often underestimated source of the neck and upper back pain [1, 3, 6]. We found that the disc degeneration exists at C7-T3; particularly 14 discs exhibited irreversible Pfirrmann grand V degeneration. In particular, the mild and moderate (grades II-III) disc degeneration could be generally seen at all levels (Table 1), and also the severe disc degeneration (grades IV-V) occurred at all levels (C7-T1: 18 discs, T1-T2: 16 discs, and T2-T3: 6 discs) and no disc at T2-T3 had grade V degeneration.

Moreover, our study further proved better accuracy of T2 mapping than MTR imaging for quantitatively grading the intervertebral disc degeneration at the cervicothoracic junction. By ROC curve analysis, T2 relaxation time values generally showed larger AUC and higher “sensitivity and specificity” than the MTR values, indicating better diagnostic ability of T2 relaxation time values. Specifically, using T2 relaxation time values, we can distinguish each two adjunct grades from each other (Figure 5), which was unable to be accomplished with MTR. Agreeing with the ROC curves, the statistical significance was detected in the comparison between each two adjunct Pfirrmann grades (P < 0.05, one-way ANOVA), while such a statistically significant difference could not be observed for the MTR values between grades II and III and between grades III and IV (Figure 4(b)) (P > 0.05). This suggests that MTR values were not competent to distinguish mild (Pfirrmann grade II), moderate (Pfirrmann grade III), and severe disc degeneration (Pfirrmann grade IV). Relevant to clinical application, the newly emerged cell-based therapies for disc degeneration are severity-dependently effective and the effect of these treatments could be monitored using quantitative MRI techniques. T2 mapping allows a clinically reliable tool to identify the appropriate candidates for the costly cell-based treatments of the intervertebral disc degeneration [26].

In addition, the T2 relaxation time value displayed a stronger correlation (r = -0.87, P < 0.0001) with the Pfirrmann grade than MTR value (r = 0.62, P < 0.0001). Such negative correlation between T2 relaxation time values and severity of disc degeneration was also previously reported [26, 31, 32]. Yoon et al. demonstrated a strong negative correlation between Pfirrmann grades and T2 relaxation time values (r = -0.742, P < 0.001) [32] at NP. Perry et al. also found that T2 relaxation time values in intervertebral discs changed with degenerative grade in a strikingly linear pattern [33]. On the other hand, Chen et al. demonstrated in a canine model that MTR values increased with the advance of the disc degeneration [34]. As a pilot study firstly comparing T2 mapping and MTR imaging at cervicothoracic junction, our data showed the stronger correlation between T2 relaxation time values and Pfirrmann grades over the ADC value, indicating the underlying advantage of T2 mapping for quantitatively evaluating the intervertebral disc degeneration to complement the clinically applied Pfirrmann grading system [31, 32].

Our study revealed no statistical difference between different genders at all three segmental levels (Table 2) and found that T2 relaxation time values and the MTR values did not vary significantly from C7-T1 to T2-T3 through one-way ANOVA (Figure 2; P = 0.25 and 0.49, resp.). Generally, the mean T2 relaxation time values of males were slightly higher than those of females, especially at T2-T3 (difference at 5.63 ms). Such tendency was consistent with our previous studies of the cervical discs [25, 26], showing shorter T2 relaxation time in females than in males without reaching statistical significance. Basically, males are easily subject to disc degeneration because of the occupational reasons and higher mechanical stress [30]. However, in the elder population, females show higher prevalence of symptomatic disc degeneration after the menopause [35].

We also found that the T2 relaxation time values decreased and the MTR value increased as the age increased. In particular, the T2 relaxation time values decreased by 38.68 ms from the age of 20-29 years to the age above 60 and the mean MTR values increased from 11.37 ± 4.35 at the age of 20-29 years to 25.98 ± 4.68 at the age above 60. Such influences of age on the T2 relaxation time value or the MTR value of the degenerated disc were previously reported by many other studies. For example, in an asymptomatic population, Niu et al. found a significant inverse correlation between the age and the T2 relaxation time of the lumbar discs [36].

Differently, all comparisons in the present study of T2 relaxation time values of different age ranges achieved statistical significance except the age ranges of 40-49 and 50-59 years. Likewise, the MTR values exhibited no significant differences when we compared MTR values of the age range of 40-49 years to either 30-39 or 50-59 years. This might be because the disc degeneration progress was relatively slower at the middle age. Notably, Mitchell and colleagues reported that middle-age individuals could regain more water diffusion after the rehabilitation activities than the young adults [37].

This study held several limitations. Firstly, this was a pilot study in a single center with a relatively small sample size and future multiple center studies with larger sample size are required to reconfirm our findings. Secondly, the radiographic findings of the present study were not confirmed by any histological and biochemical tests of biopsy samples, which also lied outside the scope of the current study. Thirdly, only symptomatic volunteers were included and the comparison with healthy controls is recommended for future investigations.

## 5. Conclusions

In conclusion, we demonstrated that T2 mapping was more accurate than the MTR MRI to quantitatively diagnose the intervertebral disc degeneration at the cervicothoracic junction. Particularly, T2 mapping allowed for precisely distinguishing disc degeneration, potentially serving as a clinical crucial tool for the emerging cell-based therapies of the intervertebral disc degeneration.

## Figures and Tables

**Table 1:** Pfirrmann grading results of all included intervertebral discs.

Pfirrmann grade	Segmental level			Total
	C7-T1	T1-T2	T2-T3	
I	17	19	21	57
II	20	21	22	63
III	12	11	18	41
IV	10	10	6	26
V	8	6	0	14
Total	67	67	67	201

**Table 2:** Comparison of the T2 relaxation time values and the MTR values between female and male patients at different anatomical levels of the cervicothoracic junction.

Anatomical level	T2 relaxation time values (ms)		P value	MTR values (%)		P value
	Female	Male		Female	Male	
C7-T1	54.69 ± 19.45	57.75 ± 18.82	0.52	19.01 ± 0.56	18.39 ± 6.83	0.72
T1-T2	56.37 ± 19.18	56.73 ± 19.06	0.99	18.98 ± 0.33	18.29 ± 6.47	0.64
T2-T3	57.84 ± 14.30	63.47 ± 15.00	0.12	16.19 ± 0.31	16.81 ± 5.48	0.28

MTR: magnetization transfer ratio; ms: millisecond. All P values were calculated by *Student's t-*test.

## Data Availability

The data used to support the findings of this study are available from the corresponding author upon request.

## Supplementary Materials

Supplementary Materials Table S1: MRI setting parameters of all MRI scan sequences including T1-weighted imaging (T1-WI), T2 weighted imaging (T2-WI), magnetization transfer (MT) imaging with and without magnetization pulse (no-MT and MT), and T2 mapping imaging. Table S2: T2 relaxation time and the MTR value between female and male patients at different anatomical levels of the cervicothoracic junction, with no significant difference of either T2 relaxation time or MTR value among different anatomical levels being detected (all P > 0.05). Table S3: comparison of T2 relaxation time and MTR value at different age, showing that, in general, T2 relaxation time decreased as the age increased, while MTR value increased with increased age. Table S4: comparison of T2 relaxation time and MTR value at different Pfirrmann grade. T2 relaxation time decreased with the advance of the Pfirrmann grade with statistically significant differences between each two adjacent grades (all P < 0.05). And the MTR value increased with higher degenerative severity, but no significant difference existed in the comparison between grades II and III and between grade III and grade IV (all P > 0.05).
