# Supplementary material for: Feasibility of T2 Mapping and Magnetic Transfer Ratio for Diagnosis of Intervertebral Disc Degeneration at the Cervicothoracic Junction: A Pilot Study
Source: Biomed Res Int. 2019 May 2;2019:6396073. doi: 10.1155/2019/6396073 (PMC6521330; doi:10.1155/2019/6396073)
Supplement: Supplementary Materials — Table S1: MRI setting parameters of all MRI scan sequences including T1-weighted imaging (T1-WI), T2 weighted imaging (T2-WI), magnetization transfer (MT) imaging with and without magnetization pulse (no-MT and MT), and T2 mapping imaging. Table S2: T2 relaxation time and the MTR value between female and male patients at different anatomical levels of the cervicothoracic junction, with no significant difference of either T2 relaxation time or MTR value among different anatomical levels being detected (all P > 0.05). Table S3: comparison of T2 relaxation time and MTR value at different age, showing that, in general, T2 relaxation time decreased as the age increased, while MTR value increased with increased age. Table S4: comparison of T2 relaxation time and MTR value at different Pfirrmann grade. T2 relaxation time decreased with the advance of the Pfirrmann grade with statistically significant differences between each two adjacent grades (all P < 0.05). And the MTR value increased with higher degenerative severity, but no significant difference existed in the comparison between grades II and III and between grade III and grade IV (all P > 0.05). [file 6396073.f1.docx]

**Table S1.** MRI setting parameters

| Sequence | T1-WI (sagittal) | T2-WI (sagittal) | T2-WI (sagittal)  NO-MT | T2-WI (sagittal)  MT | T2 mapping (sagittal) |
| --- | --- | --- | --- | --- | --- |
| Repetition (ms) | 571 | 2580 | 3000 | 3000 | 1500 |
| Echo time (ms) | 9.5 | 101 | 96 | 96 | 8.5 – 67.9 |
| Field of view (mm) | 24 × 24 | 24 × 24 | 26 × 26 | 26 × 26 | 20 × 20 |
| Matrix (mm × mm) | 320 × 240 | 320 × 240 | 320 × 240 | 320 × 240 | 256 × 160 |
| Slice thickness (mm) | 4 | 3 | 4 | 4 | 3 |
| Interslice gap (mm) | 0.4 | 0.3 | 0.4 | 0.4 | 0.6 |
| Number of slices | 7 | 10 | 8 | 8 | 64 |
| Echo trains/slice | 4 | 15 | 21 | 21 | - |
| Band width (KHz) | 240 | 283 | 284 | 284 | 31.25 |
| Offset (HZ) | _ | _ | _ | 1100 | _ |
| Number of signal-intensity acquisition | 2 | 2 | 2 | 2 | 1 |
| Examination time (s) | 120 | 172 | 138 | 138 | 247 |

ms: milliseconds; mm: millimeter; KHz: Kilo Hertz; s: second; T1-WI: T1-weighted imaging; T2-WI: T2-weighted imaging;

**Table S2.** Comparison of T2 relaxation time and MTR value at different age

| Age range | Number of discs | T2 relaxation time (ms) | MTR value (%) |
| --- | --- | --- | --- |
| 20 - 29 | 57 | 75.78 ± 13.44 **^‡ ¶ # &^** | 11.37 ± 4.35 **^‡ ¶ # &^** |
| 30 - 39 | 24 | 65.20 ± 8.52 **^† ¶ # &^** | 16.38 ± 2.20 **^† # &^** |
| 40 - 49 | 42 | 54.86 ± 8.35 **^† ‡ &^** | 18.65 ± 3.00 **^† &^** |
| 50 - 59 | 36 | 52.06 ± 8.35 **^† ‡ &^** | 20.47 ± 2.01 **^† ‡ &^** |
| ≥ 60 | 42 | 37.10 ± 10.70 **^† ‡ ¶ #^** | 25.98 ± 4.68 **^† ‡ ¶ #^** |

MTR: magnetization transfer ratio; ms: millisecond; ^†^ P < 0.05 compared with discs of age range 20-29. ^‡^P < 0.05 compared with discs of age range 30-39; ^¶^P < 0.05 compared with discs of age range 40-49; ^#^P < 0.05 compared with discs of age range 50-59; ^&^P < 0.05 compared with discs of age ≥ 60.

**Table S3.** Comparison of T2 relaxation time and MTR value at different Pfirrmann grade

| Pfirrmann grade | T2 relaxation time (ms) | MTR value (%) |
| --- | --- | --- |
| I | 74.66 ±12.25 **^‡ ¶ # &^** | 13.93 ± 5.07 **^‡ ¶ # &^** |
| II | 63.99 ± 8.15 **^† ¶ # &^** | 17.65 ± 5.20 **^† # &^** |
| III | 49.88 ± 5.68 **^† ‡ # &^** | 19.11 ± 4.15 **^† &^** |
| IV | 36.43 ± 5.87 **^† ‡ ¶ &^** | 22.24 ± 5.17 **^† ‡ &^** |
| V | 25.37 ± 3.85 **^† ‡ ¶ #^** | 29.00 ± 4.09 **^† ‡ ¶ #^** |

MTR: magnetization transfer ratio; ms: millisecond. ^†^P < 0.05 compared with discs of Pfirrmann grade I; ^‡^P < 0.05 compared with discs of Pfirrmann grade II; ^¶^P < 0.05 compared with discs of Pfirrmann grade III; ^#^P < 0.05 compared with discs of Pfirrmann grade IV; ^&^P < 0.05 compared with discs of Pfirrmann grade V.

**Table S4.** Receiver operating characteristic (ROC) curves to compare the diagnostic accuracy of T2 mapping and MTR for the discrimination between each two consecutive Pfirrmann grades

| Pfirrmann grade | T2 relaxation time | | | |  | MTR value (%) | | | |
| --- | --- | --- | --- | --- | --- | --- | --- | --- | --- |
|  | Cut-off value  (ms) | AUC | Sensitivity% | Specificity% |  | Cut-off value | AUC | Sensitivity% | Specificity% |
| I *versus* II | 77.12 | 0.77 | 96.83 | 47.37 |  | 15.96 | 0.69 | 69.84 | 61.40 |
| II *versus* III | 54.60 | 0.92 | 85.37 | 90.48 |  | 12.17 | 0.58 | 97.57 | 17.46 |
| III *versus* IV | 45.45 | 0.97 | 100.00 | 80.49 |  | 17.24 | 0.67 | 88.46 | 41.46 |
| IV *versus* V | 31.70 | 0.94 | 100.00 | 80.77 |  | 23.78 | 0.84 | 100.00 | 65.38 |

MTR: magnetization transfer ratio; AUC: area under curve; ms: millisecond.
